# The impact of volume expansion on thermodynamic and kinetic properties of graphite/Si alloy composite anodes

**DOI:** 10.1039/d5ra07317k

**Published:** 2025-12-04

**Authors:** Min-ho Lee, Orynbassar Mukhan, Carlos Tafara Mpupuni, Batukhan Tatykayev, Zhumabay Bakenov, Sung Soo Kim

**Affiliations:** a Graduate School of Energy Science and Technology, Chungnam National University 99 Daehak-ro Yuseong-gu Daejeon 34134 Republic of Korea kimss@cnu.ac.kr; b Institute of Batteries LLC Kabanbay Batyr Ave. 53 Astana 010000 Kazakhstan; c National Laboratory Astana, Nazarbayev University Kabanbay Batyr Ave. 53 Astana 010000 Kazakhstan; d Department of Chemical and Materials Engineering, School of Engineering and Digital Sciences, Nazarbayev University Kabanbay Batyr Ave. 53 Astana 010000 Kazakhstan

## Abstract

With the growing demand for high energy density and fast-charging/high-rate performance, understanding the thermodynamic and kinetic behavior of graphite/silicon composite anodes has become increasingly important. In this study, graphite/Si alloy composite electrodes containing 0–50 wt% silicon alloy were investigated using a three-electrode pouch cell combined with *in situ* dilation. Through this analysis, the changes in porosity after the formation cycles were determined, while the lithium-ion diffusion coefficient (*D*_Li^+^_) was calculated based on the Weppner–Huggins equation. In addition, the resistance components were quantitatively evaluated by pulse polarization and electrochemical impedance spectroscopy (EIS). This study elucidates the effect of porosity evolution, induced by irreversible expansion with varying silicon content, on the electrode's electrochemical behavior. For the YNG-30% electrode, irreversible expansion during the formation process increased the porosity from 32.3% to 55.0%. As a result, the transport pathways for solvated Li^+^ were expanded, and the charge-transfer resistance (*R*_ct_) decreased, leading to enhanced interfacial and interparticle diffusion in the graphite staging region. In contrast, in the silicon-dominant region, intrinsic thermodynamic and kinetic limitations resulted in higher overpotential at the beginning of charge and the end of discharge. Consequently, the role of silicon is not limited to increasing capacity but serves as a structural design element for achieving fast-charging and high-rate performance. These findings highlight that porosity optimization is a key factor for improving electrode performance.

## Abstract 2

Irreversible expansion induced by silicon increases electrode porosity, thereby enhancing thermodynamic and kinetic properties through improved lithium-ion diffusion and charge-transfer within the graphite reaction region.

## Graphical abstract

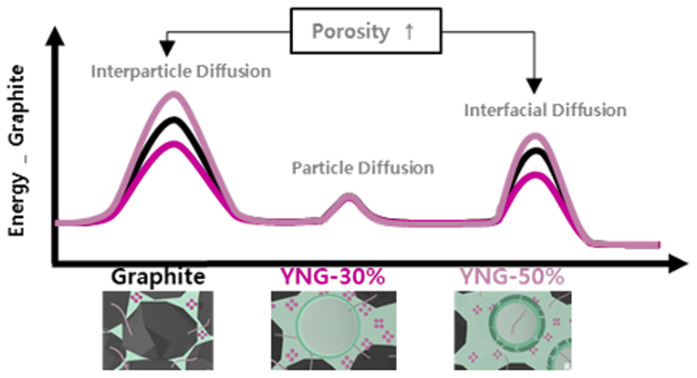

## 1 Introduction

Lithium-ion batteries (LIBs) have become critical energy storage devices due to their widespread use in electric vehicles, portable electronics, and large-scale energy storage systems, resulting in increasing demand for higher energy density.^1^

Graphite anodes are still the most widely used anode materials due to their low cost, abundant resources, and stable cycle life.^2^ During charge and discharge, lithium-ions are inserted into and extracted from the graphite structure, forming graphite intercalation compounds (GICs).^3^ The GICs are classified into different stages depending on the amount of lithium intercalation between graphene layers. For instance, stage 1 corresponds to the fully lithiated state where lithium-ions occupy every interlayer, while stage 2 indicates a structure in which lithium-ions are inserted between every second graphene layer.^4^ Nevertheless, the limited theoretical capacity of graphite has prompted extensive research on alternative anode materials.

Among various anode candidate materials, silicon has attracted significant attention as a next-generation material due to its high theoretical capacity of 4200 mAh g^−1^ for Li_4.4_Si, which is over ten times higher than that of graphite (372 mAh g^−1^ for LiC_6_).^5^ However, the severe volume expansion of silicon during lithiation/de-lithiation has been shown to cause pulverization, loss of electrical contact and unstable SEI formation, leading to poor cycle stability.^6^ To address issues of cycle stability and low conductivity, a wide range of silicon-based materials has been researched. These materials include nano-sized silicon,^7,8^ a silicon carbon composite (Si/C),^9,10^ silicon oxide (SiO_*x*_),^11,12^ and porous silicon.^13,14^ Among these, silicon alloys have been studied extensively because the inactive matrix suppresses volume expansion and improves conductivity. Our previous study demonstrated that amorphous silicon undergoes recrystallization into a crystalline phase during heat treatment, leading to the uniform distribution of nano-sized silicon throughout the inactive matrix. This structural evolution effectively mitigates volume changes and enhances both mechanical stability and electrochemical performance.^15–18^ Nevertheless, the practical application of silicon-based single electrodes remains challenging. Consequently, for the purpose of practical commercialization, they are combined with graphite, typically at a ratio of up to 5 wt%.^19,20^ This graphite/silicon composite anode facilitates high energy density and excellent cycle life characteristics. This can be attributed to the fact that graphite has the ability to mitigate silicon's volume expansion and to provide electrical conductivity.^21,22^ Despite these advantages, research on the electrochemical behavior of Si/graphite composite anodes, such as overpotential and diffusivity, remains insufficient. Dominik Wycisk *et al.* reported that the pronounced overpotentials observed in silicon-containing electrodes at low current densities originate mainly from mechanical stress, indicating that mechanical effects need to be considered alongside transport limitations in the analysis of Si anodes.^23^ Tuan Kiet Pham *et al.* has reported the analysis of lithium-ion diffusivity in graphite/silicon composite anodes using the Weppner–Huggins equation, and highlighted that the correlation between Si nanoparticle content, overpotential, and diffusion coefficients is strongly influenced by the staging behavior of graphite. Their study demonstrated a clear interplay during lithiation and de-lithiation, where graphite governs phase transitions, while Si nanoparticles dominate under liquid-like Li^+^ diffusion.^24^ However, this analysis has limitations as it does not account for changes in electrode thickness (*L*) occurring during the cycling process. For pure graphite electrodes, the 10–15% volume expansion during lithiation does not significantly impact the analysis. However, when silicon is mixed, larger volume expansion occurs, and irreversible expansion also happens after discharge. Therefore, failing to account for this can lead to calculated diffusivity values lower than the actual value, resulting in underestimation. Consequently, reflecting the electrode thickness change is necessary for deriving a more accurate diffusivity.

*In situ* dilation has been employed since the 1970s to measure the expansion that occurs when ions, atoms, or molecules are inserted into the interlayers of host compounds. This technique is useful for analyzing electrode degradation, as it allows direct observation of the volume change characteristics during lithiation/de-lithiation, including both reversible and irreversible expansion upon cycling.^25^ In the case of graphite, dilation behavior has been combined with *in situ* XRD to elucidate structural transitions and volume change features during lithiation/de-lithiation, and has also been widely applied in studies investigating the structural stability and porosity of electrode.^26–29^

Addressing this need, this study simultaneously measured *in situ* dilation and employed the galvanostatic intermittent titration technique (GITT) for graphite/silicon composite electrodes with varying silicon contents, systematically analysing changes from thermodynamic parameters (overpotential, open-circuit voltage (OCV)), kinetic parameters (*D*_Li^+^_, charge-transfer resistance, exchange current), and electrode structural evolution (porosity). In particular, the use of a three-electrode pouch cell allowed direct measurement of anode potential and real-time monitoring of volume expansion, thereby complementing the limitations of the conventional Weppner–Huggins-based analysis and enabling a more accurate determination of the lithium-ion diffusion coefficient. Furthermore, by performing pulse polarization process, the resistance components of the electrode were quantitatively evaluated. Changes in electrode porosity after formation cycles were calculated, clarifying the correlation between electrode structural evolution and electrochemical behavior. Through this approach, the study provides a comparative analysis with previous reports that associated silicon with adverse effects such as increased overpotential, reduced diffusivity, and electrode degradation, demonstrating instead that the porosity evolution of the electrode can exert a positive influence on its electrochemical behavior. In particular, this study newly proposes that the role of silicon is not limited to simply contributing capacity or reducing diffusion distance under identical loading conditions, but that the change in porosity formed by irreversible expansion is a key factor enhancing diffusion behavior and charge transfer during fast charging and high-power operation. Overall, this combined analytical approach provides a more comprehensive framework for understanding graphite/silicon composite anodes and may offer useful guidelines for designing electrodes with fast-charging and high-rate performance.

## 2 Experimental section

### 2.1 Materials and electrode preparation

For the preparation of anode electrodes, two types of active materials were employed: natural graphite and Si alloy powders. Natural graphite (BTR New Energy Materials Inc.) was used without further treatment. High-grade elements (>99.9%) of Si, Al, Fe, Ni, and Cu were acquired from Taewon Scientific Co., Ltd. An amorphous Si alloy ribbon was synthesized using a cost-effective and scalable melt-spinning technique under a high-purity Ar atmosphere. The alloy composition was selected as Si (53 at%), Al (29 at%), Fe (15 at%), Mn (2 at%), and Cu (1 at%). Details of the equipment and synthesis process have been reported in our previous studies.^16–18^ The ribbons obtained from the melt-spinning process were mechanically crushed in *n*-hexane using a paint shaker (Nara Science Corp., KM-2000T) with Zr balls at a ball-to-powder weight ratio of 1 : 40 for about 15 min. The amorphous powders were then crystallized by heat treatment in a tube furnace (Ajeon Heating Industrial Co., Ltd) at 873 K for 60 min with a ramp rate of 3 K min^−1^ under an Ar atmosphere, as confirmed by DSC results. These two active materials were mixed at different weight ratios (0, 10, 20, 30, 40, 50% Si alloy) to prepare composite anodes.

The anode slurry was prepared by mixing 97 wt% active materials (graphite and Si alloy with varying ratios from 0 to 50%, denoted as graphite, YNG-10% to YNG-50%), 0.5 wt% single-walled carbon nanotubes (SWCNTs), 1.5 wt% carboxymethyl cellulose (CMC), and 1.0 wt% styrene-butadiene rubber (SBR). The components were homogenized using a planetary mixer (at 2000 rpm, Thinky, Japan). The resulting slurry was coated onto a Cu foil using a doctor blade with a thickness of ∼8 µm and then dried in a vacuum oven at 110 °C for 6 h. The mass loading of the anode electrodes was controlled in the range of 3.8–4.4 mg cm^−2^, and the electrode density was adjusted to 1.6 g cm^−3^, as summarized in Table S1. The cathode slurry was composed of 96 wt% LiNi_0.6_Co_0.2_Mn_0.2_O_2_ (NCM622) as the active material, 2 wt% Super P as the conductive agent, and 2 wt% polyvinylidene fluoride (PVDF) as the binder in *N*-methyl-2-pyrrolidone (NMP) solvent. The slurry was coated on an Al foil and subsequently dried in a vacuum oven at 90 °C for 6 h. For full-cell assembly, the prepared anodes and cathodes were paired with an N/P ratio of 1.05. In addition, a LiFePO_4_ (LFP) electrode was used as the reference electrode, which was fabricated using 96 wt% active materials, 2 wt% Ketjen black, and 2 wt% CMC, coated on an Al foil. The LFP electrodes were also dried at 90 °C under vacuum for 6 h.

### 2.2 Materials characterization

The crystallization behavior of the Si alloy powders was analyzed using differential scanning calorimetry (DSC, Netzsch 404) with a heating rate of 10 K min^−1^ under an Ar atmosphere. The crystal structures were examined by X-ray diffraction (XRD, Rigaku SmartLab). The microstructure was examined by a spherical aberration corrected scanning transmission electron microscopy (Cs-STEM, JEM-ARM200F, JEOL). The true densities (*ρ*_i_) of the materials, required for porosity calculation, were measured using a helium pycnometer (AccuPyc II 1340).

### 2.3 Electrochemical characterization

Coin-type half cells and full cells (CR2032, Welcos Co., Ltd) were assembled with the as-prepared electrodes, a microporous polyethylene (PE, W-Scope Korea) separator, and an electrolyte of 1 M LiPF_6_ in ethylene carbonate (EC)/ethyl methyl carbonate (EMC) (3 : 7, v/v; Dongwha Electrolyte Co., Ltd) containing 5 wt% fluoroethylene carbonate (FEC). For half cells, lithium metal foils (Rockwood Lithium) were used as counter/reference electrodes, while for full cells, the as-prepared LiNi_0.6_Co_0.2_Mn_0.2_O_2_ (NCM622) cathode was employed as the counter electrode. All cell assembly was carried out in an Ar-filled glove box.

Before electrochemical testing, all cells were subjected to a formation cycle following a 12 h resting period. The formation process was carried out for three cycles at 25 °C, consisting of constant current/constant voltage (CC/CV) charging at 0.1 C with a cut-off current of 0.05 C, and discharging at 0.1 C within a voltage range of 0.005–2.0 V.

During both charge and discharge processes, pulse currents were applied at designated states of charge (SOC = 10, 30, 50, 70, and 90%) and depth of discharge (DOD = 10, 30, 50, 70, and 90%). The applied currents ranged from 0.025 C to 10 C and were maintained for 10 s. After each pulse, the cells were rested for 2 h, and the voltage measured after this period was defined as the OCV. The experimental procedure has been described in our previous work.^30^

### 2.4 *In situ* electrochemical dilatometry measurement

A single-layer pouch cells were assembled in a dry room using the same configuration, in which the previously prepared YNG anode and LiNi_0.6_Co_0.2_Mn_0.2_O_2_ (NCM622) cathode were used. A LiFePO_4_ (LFP) electrode, exhibiting a stable potential as a reference electrode, was introduced as the reference electrode for anode potential monitoring.^31,32^ A LFP electrode, prepared by disassembling a cell charged to 0.1 C for 5 h, served as the reference electrode. As confirmed in Fig. S1(a), the LFP reference exhibited stable potential retention over long durations. Additionally, the reference electrode was positioned to minimize the anode overhang and reduce resistance, as shown in Fig S1(b).^33^

The *in situ* dilation setup, designed in-house, was employed to monitor the real-time volume change of the electrodes. Single-pouch cells prepared as described above were connected to an Espec (US) cycler and software for simultaneous recording of electrode thickness and anode potential using an LFP reference electrode. At this time, the gap sensor used in the dilatometer had a resolution of 0.3 µm, and the internal temperature was maintained at 24 °C. Galvanostatic measurements were conducted for three cycles within the voltage range of 4.25–2.5 V at 0.1 C (CC/CV charging with a cut-off current of 0.05 C and CC discharging). After the formation cycles, the pouch cells were subjected to GITT measurements, consisting of 30 min charge pulses at 0.1 C followed by a 2 h rest period. The Li^+^ diffusion coefficients were calculated using the Weppner and Huggins equation based on Fick's second law, by incorporating the measured thickness changes and the OCV obtained after each rest step. EIS (IVIUM) was conducted in the discharged state after three formation cycles, within a frequency range of 10^6^–10^−2^ Hz and an AC amplitude of 10 mV.

## 3. Result and discussion

### 3.1 Characterization of Si alloy

**Fig. 1:**
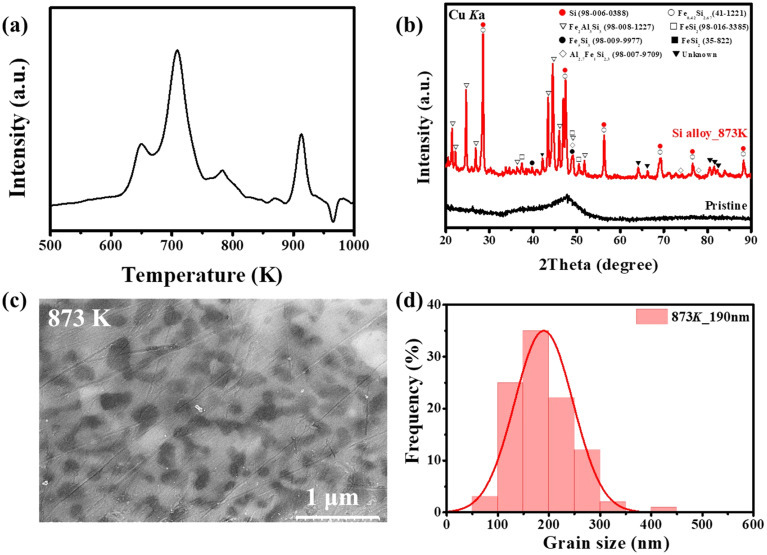
(a) DSC curve of as-prepared amorphous Si alloy. (b) XRD patterns of pristine and annealed Si alloy at 873 K. (c) TEM image of annealed Si alloys. (d) Grain size distribution of Si crystallites obtained from TEM images.

The Si alloy ribbons used in this study were synthesized *via* the cost-effective and scalable melt-spinning technique. The alloy composition is Si (53 at%), Al (29 at%), Fe (15 at%), Mn (2 at%), Cu (1 at%), a glass-forming alloy designed based on the empirical rules of Inoue *et al.*^34,35^ The thermal behavior of the alloy was confirmed *via* DSC analysis (Fig. 1(a)). A distinct exothermic peak appeared in the 800–873 K range, corresponding to a recrystallization reaction accompanied by decomposition of the supersaturated solid solution and formation of intermetallic compounds. Accordingly, heat treatment was performed at 873 K. XRD analysis (Fig. 1(b)) revealed that an amorphous structure dominated before heat treatment. After heat treatment, diffraction peaks of active Si were clearly observed alongside intermetallic compounds such as FeSi_2_ and Al_3_Fe_2_Si_3_. This indicates that Si grain growth and phase separation occurred during the heat treatment process. TEM observation (Fig. 1(c)) confirmed that crystalline Si grew to hundreds of nanometers in size and was uniformly distributed. The grain size distribution (Fig. 1(d)) ranged from approximately 100–500 nm, with an average of about 188 nm. These results indicate that the heat treatment caused the active Si grains to grow and the phase separation from the alloy matrix to become distinct. The uniformly distributed nano-sized Si and inactive matrix can contribute to suppressing volume expansion and enhancing electrical conductivity. Furthermore, the initial voltage profile observed in Fig. S3 accurately reflects the reaction of crystalline Si confirmed by XRD. The initial charge/discharge capacities of the single electrode were 932.9/836.1 mAh g^−1^, respectively, and it exhibited a capacity retention rate of 74.4% even after 100 cycles. These results were subsequently utilized as a basis for graphite/Si composite anode research.

### 3.2 Electrochemical analysis using three-electrode full cell with *in situ* dilation

**Fig. 2:**
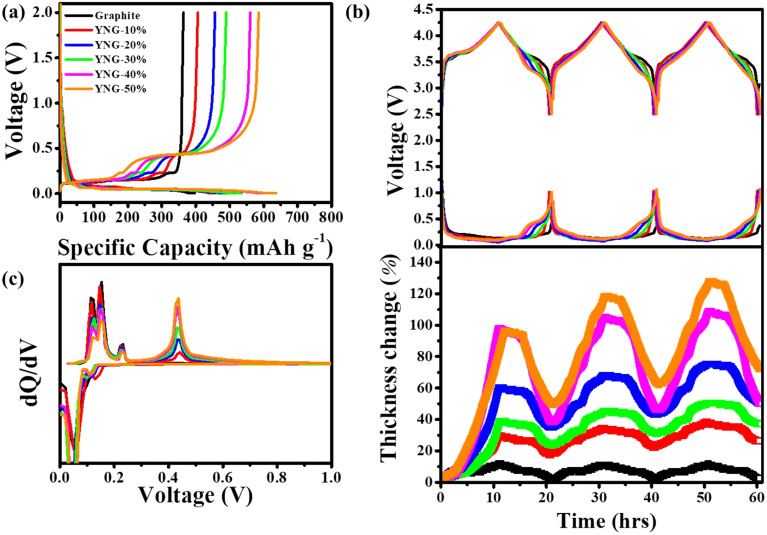
(a) Initial charge–discharge voltage profiles of graphite/silicon composite anodes with varying silicon contents. (b) *In situ* electrochemical dilatometry (ECD) measurement results over three formation cycles of the assembled single-layer pouch full cells, along with the corresponding full-cell voltage and anode potential. (c) d*Q* d*V*^−1^ curves of the composite anodes.

Fig. 2(a) and (c) show the initial charge–discharge profiles and the corresponding d*Q* d*V*^−1^ curves of electrodes with different Si alloy contents. As the Si content increases, both the capacity and the overall shape of the curves change significantly. The d*Q* d*V*^−1^ analysis reveals distinct reaction behaviors during lithiation and de-lithiation, which can be attributed to the distinct reaction characteristics of the graphite/Si composite anodes. Graphite typically begins lithiation at approximately 0.20 V; thus, the voltage range of 1.0–0.20 V corresponds mainly to Si lithiation, while 0.20–0.01 V represents the region where both Si and graphite lithiate simultaneously. In contrast, during de-lithiation, the voltage range of 0.01–0.23 V is primarily associated with the de-lithiation of graphite, whereas 0.23–1.0 V corresponds to the de-lithiation of Si.^36,37^ Consequently, as the Si content increases, the peak intensity associated with graphite phase transitions during de-lithiation diminishes, while the c-Li_15_Si_4_ de-lithiation peak increases in alignment with the Si fraction. These differences in the reaction regions during charge and discharge can serve as the underlying cause for the variations in electrochemical behavior, such as overpotential and diffusivity, observed in the electrodes.

Fig. 2(b) presents the results obtained from three-electrode single-layer pouch full-cells, where the graphite/Si alloy composite electrode was used as the anode, LFP as the reference electrode, and NCM622 as the cathode. The plots show the full-cell voltage, anode potential, and electrode thickness variation as a function of time, with the composition and characteristics of each anode summarized in Table. S1. All cells underwent formation cycles prior to GITT measurements, and the initial conditions were carefully controlled to be as similar as possible. For the graphite electrode, a volume expansion of approximately 10.5% was observed during charging, which remained nearly constant over the subsequent three cycles. The irreversible expansion was approximately 1.7% in the first and second cycles and 2.5% in the third cycle, indicating that the electrode volume change was highly stable and reversible. Such stage-dependent expansion and contraction behaviors are consistent with previous literature, thereby validating the reliability of the measurement method employed in this study.^26,27,38,39^ The NCM cathode exhibited only about 2% expansion, suggesting that its contribution to overall cell swelling was negligible.^39^ Since it is difficult to clearly distinguish the volume changes of the anode and cathode in a full cell, this study was conducted by excluding the influence of cathode expansion.^28,40^

As the Si content increased, both the charging-induced expansion and the residual irreversible expansion after discharge became larger, and this tendency became more apparent with cycling. The YNG-50% electrode showed the largest extent of swelling, with an expansion of 96.3% in the first cycle and 127.7% in the third cycle. Even after discharge, 72.4% of irreversible expansion remained. Typically, graphite electrodes exhibit stepwise expansion and contraction depending on the stages. However, this feature was reduced when Si was introduced, confirming that the large expansion and irreversible swelling observed were mainly due to the Si contribution.^29^ As a result, the electrode exhibited residual swelling even after discharge, which can be regarded as an important indicator related to the structural stability of the electrode. As a result, the electrode exhibited residual swelling even after discharge, which can be regarded as an important indicator related to the structural stability of the electrode. This irreversible volume change mainly originates from the formation of irreversible SEI layers, the rearrangement of particles within the electrode during expansion, and delamination, all of which can strongly influence the electrode stability.^27^ However, such irreversible expansion can also affect the electrode porosity,^28,29^ and in some cases, may even play a beneficial role in electrochemical performance.

### 3.3 Overpotential and volume change

**Fig. 3:**
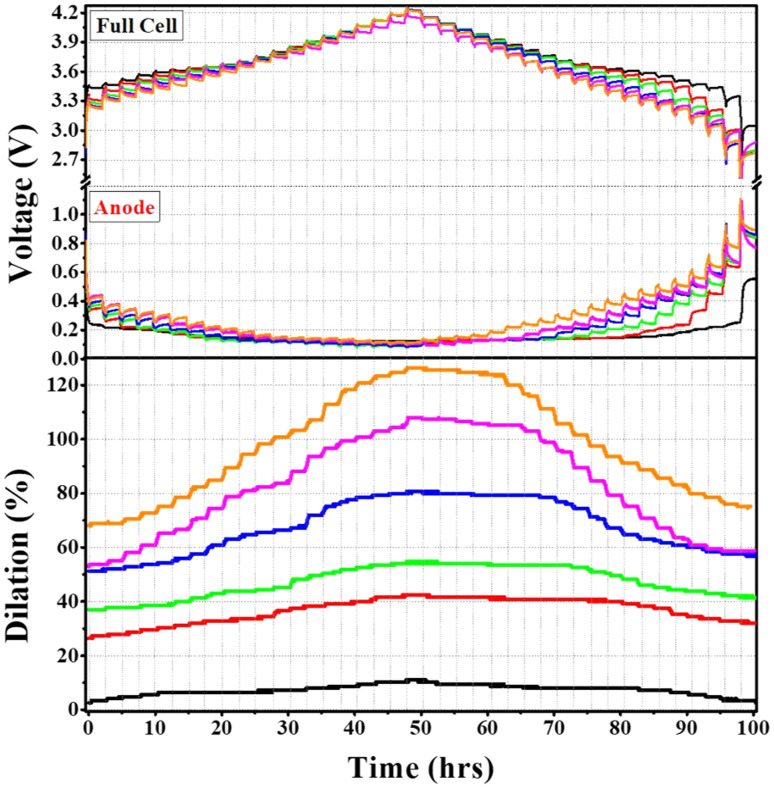
GITT profiles after three formation cycles at 0.1 C, showing full-cell voltage, anode potential, and corresponding volume changes.

**Fig. 4:**
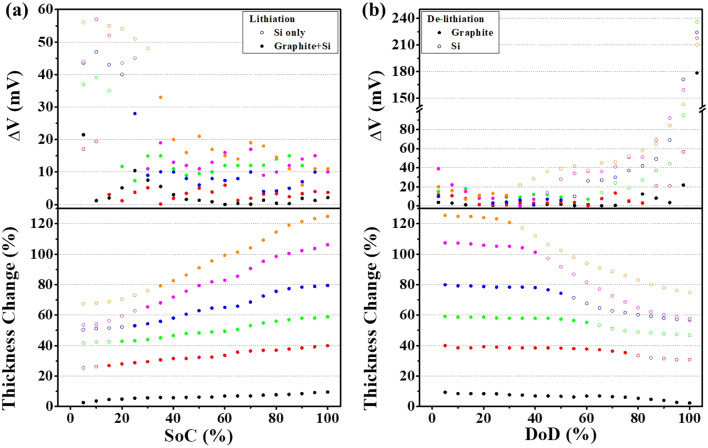
Δ*V* (the voltage difference between constant-current/constant-voltage (CCV) charging and open-circuit voltage (OCV)) and volume change as a function of (a) SOC and (b) DOD.

Fig. 3(a) and (b), shows the time behavior of the GITT measurement conducted at 0.1 C after three formation cycles, while Fig. 4(a) and (b) present the behavior of overpotential and volume change as a function of SOC and DOD, respectively. In this study, the overpotential was defined as the difference between the cell potential at the end of each current pulse and the OCV measured after a 2-hours rest time.

During the lithiation process, the overpotential was initially high and then gradually decreased as the SOC increased. Conversely, during the de-lithiation process, the overpotential was low at the beginning and increased significantly at the end of discharge. As explained earlier, this phenomenon can be attributed to the different reaction characteristics of the graphite/Si composite anode. At the early SOC region, the graphite electrode already exhibits a relatively high overpotential due to sluggish Li diffusion and the pronounced concentration gradient formed at the electrode surface. This phenomenon is attributable to the reduced activation of the electrochemical reaction during the initial phase of the SOC, which leads to elevated charge transfer resistance.^41^ The initial overpotential is further increased by the inherently low conductivity and sluggish reaction kinetics of amorphous silicon. In particular, within the silicon-only lithiation region, these limitations combined with the mechanical stress generated during lithiation lead to a more pronounced increase in overpotential.^23,42^ When Si and graphite are simultaneously lithiated, the volume expansion of Si enhances interparticle contact and extends conductive pathways, thereby reducing the overpotential. In contrast, once graphite lithiation is completed, the Si-only reaction region again exhibits an increase in overpotential, with the magnitude of this increase becoming more pronounced as the Si content rises.^42^ During de-lithiation, as previously described, the reaction regions of graphite and Si were clearly separated, with lithium-ion extraction occurring first from graphite followed by silicon. In the graphite-only region, a consistently low overpotential and small volume change were observed, whereas once Si de-lithiation commenced, the overpotential gradually increased and the volume change became more significant. Interestingly, as shown in Fig. 4(a) and (b), the YNG-30% electrode, despite its relatively high Si content and irreversible expansion, exhibited a lower overpotential than YNG-20%, 40%, and 50% both in the region where Si and graphite undergo lithiation simultaneously and in the graphite de-lithiation region. This trend was also confirmed in Fig. S4, where the same behavior was observed at an identical OCV state. Such behavior is closely related to the overall resistance of the electrode, highlighting the need to identify the contributing factors. Overpotential is generally divided into ohmic resistance, charge-transfer resistance, and diffusion resistance.^43^ Therefore, in this work, the relative contributions of these overpotentials will be further examined and quantitatively confirmed through subsequent analyses of lithium-ion diffusivity and resistance.

### 3.4 Diffusivity

**Fig. 5:**
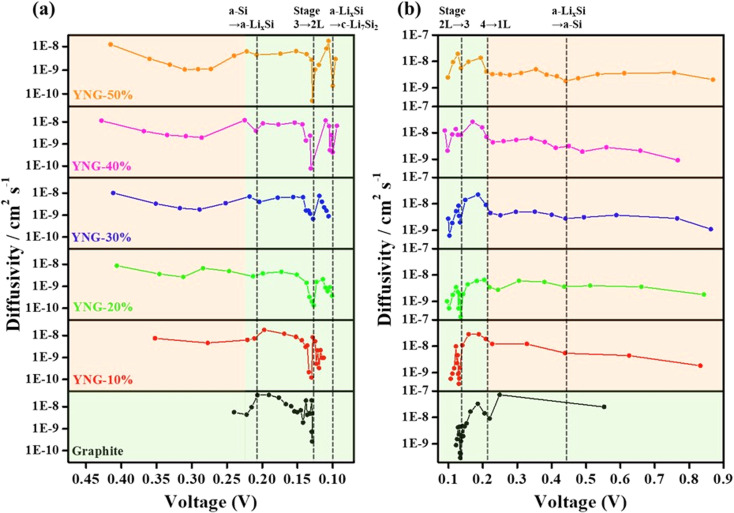
GITT-derived lithium-ion diffusion coefficients of graphite and YNG anodes (10–50% Si) during (a) lithiation and (b) de-lithiation, calculated by the Weppner–Huggins equation based on Fick's second law. Red boxes denote Si-only reaction regions, while green boxes indicate graphite-dominant or co-reaction regions.

The lithium-ion diffusion coefficients were calculated from GITT measurements using the Weppner–Huggins equation derived from Fick's second law,^44^ as follows:1
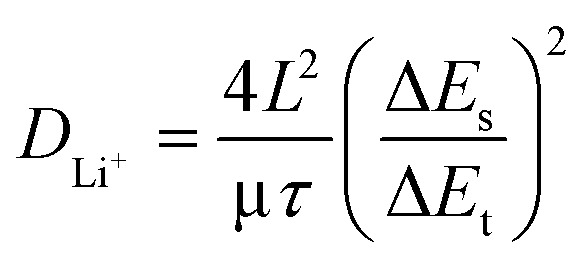
In this equation, *τ* is the current pulsing time (*s*). Δ*E*_t_ the potential change during the current pulse (excluding the iR drop), and Δ*E*_s_ is the steady-state voltage. *L* is the electrode thickness (cm), obtained from *in situ* dilation so that the precise thickness state of the electrode was considered in the calculation. Fig. 5(a) and (b) present the diffusion coefficients of each electrode during charge and discharge. Lithium-ion diffusion is a key factor in optimizing electrode performance, as insufficient diffusion leads to concentration gradients at the electrode surface and consequently causes overpotential.^45,46^ For graphite electrodes, the thickness change upon lithiation was within approximately 10%, thus having a comparatively negligible effect on the calculation. Conversely, the YNG-50% electrode exhibited an expansion of approximately 120% at 100% SOC, which could result in up to a fourfold deviation in the calculated diffusion coefficient. Therefore, in this work, the electrode thickness was corrected using *in situ* dilation measurements to obtain more accurate diffusion coefficients.

During lithiation of graphite, the diffusion coefficient was initially low (0.25–0.20 V), which can be attributed to the insignificant change in the interlayer spacing. At this stage, a large overpotential is observed in both lithiation and de-lithiation. As the *d*-spacing expands more significantly, sufficient pathways for lithium-ion transport are created, leading to an increase in the diffusion coefficient.^47,48^ In the region where the LiC_24_ phase is formed, the diffusion coefficient decreases again because lithium-ions are intercalated in a more compact manner, which reduces their mobility. When the transition from LiC_24_ to LiC_12_ occurs, the diffusion coefficient rises once more, as lithium is more fully intercalated and can diffuse from the edge planes to the basal planes, accompanied by a larger entropy contribution.^48,49^ A subsequent sharp drop in the diffusion coefficient appears before the formation of the LiC_6_ phase, which is attributed to the formation of superdense intermediate phases such as Li_7_C_24_ and Li_11_C_24_. These phases generate a lithium concentration gradient between the particle surface and the interior, thereby suppressing diffusion.^48^ A similar trend is observed during de-lithiation. The diffusivity of graphite ranged from 2.65 × 10^−10^ cm^2^ s^−1^ ∼ 3.3 × 10^−8^ cm^2^ s^−1^ during lithiation and from 2.30 × 10^−10^ cm^2^ s^−1^ ∼ 7.26 × 10^−10^ cm^2^ s^−1^ during delithiation, which is similar to previous results.^50,51^ In graphite/silicon composite anodes, previous studies have reported that graphite governs the lithium-ion diffusion behavior during phase transition processes, whereas silicon dominates in the liquid-like diffusion regime. Therefore, the overall lithium-ion diffusion in Si–graphite composite electrodes can be understood as a balance between the contributions of graphite and Si, largely following the behavior of graphite but either enhanced or suppressed depending on the Si content.^24^

During lithiation, all YNG electrodes exhibited low diffusivity in the silicon reaction region. This decrease was observed around 0.26 V, corresponding to the transition from amorphous Si (a-Si) to a-Li_*x*_Si. A further decrease in diffusivity was observed near 0.09 V, which is associated with the transition from a-Li_*x*_Si to c-Li_7_Si_2_. In graphite, the reduction in diffusivity caused by phase transitions mainly appeared during the transition from stage 3 to 2 L. In other words, in the staging region, the diffusion behavior is primarily governed by graphite, whereas in the 2 L region it is dominated by the phase transition of silicon from a-Li_*x*_Si to c-Li_7_Si_2_.^24^ In most cases, diffusivity in the graphite staging region decreased with higher silicon content; however, the YNG-30% electrode exhibited an unusual improvement in diffusivity. Around 0.13 V, corresponding to the graphite staging transition (stage 3 → 2 L), the *D*_Li^+^_ of the graphite electrode was 2.65 × 10^−10^ cm^2^ s^−1^. For silicon-containing composites (YNG-10 to 50%), *D*_Li^+^_ varied as follows: YNG-10% (1.23 × 10^−10^ cm^2^ s^−1^), YNG-20% (1.37 × 10^−10^ cm^2^ s^−1^), YNG-30% (6.70 × 10^−10^ cm^2^ s^−1^), YNG-40% (7.87 × 10^−11^ cm^2^ s^−1^), and YNG-50% (5.10 × 10^−11^ cm^2^ s^−1^). Among these, YNG-30% exhibited the highest diffusivity in the graphite staging region. In the silicon-dominant region (0.22–0.24 V), *D*_Li^+^_ ranged between 3.46 × 10^−9^ and 6.21 × 10^−9^ cm^2^ s^−1^, showing only minor variation across silicon contents. The diffusion coefficient is generally influenced by the material itself and the electrode structure.^46,52^ Weng *et al.* classified the diffusion behavior in graphite electrodes into interfacial diffusion, particle diffusion, and electrode-level (interparticle) diffusion. The diffusion behavior of graphite described earlier can largely be understood as particle diffusion associated with changes in interlayer spacing during phase transitions. However, the overall Li^+^ transport within the electrode is also influenced by interparticle and interfacial diffusion, whose roles become more significant in graphite/silicon composite anodes. This is because the incorporation of silicon induces more pronounced structural changes in the electrode, thereby modifying interparticle pathways and interfacial characteristics. In particular, interparticle diffusion is strongly affected by the electrode porosity, as an appropriate porosity provides channels through which solvated Li^+^ ions can migrate. As a result, the impact of interparticle and interfacial diffusion on the measured diffusion coefficient becomes more evident.^52^ Therefore, the relatively high diffusivity observed in YNG-30% is attributed to apparent effects arising from electrode structural evolution. In contrast, the consistently low diffusivity in the initial silicon reaction region originates from the intrinsic characteristics of silicon, namely its inherently low conductivity and sluggish ion transport.^53^ During de-lithiation, when silicon dominates the reaction, all electrodes showed similarly low diffusivity. However, as the silicon content increased, a gradual increase in *D*_Li^+^_ was observed during the phase transition of graphite. This trend correlates with the d*Q* d*V*^−1^ results in Fig. 2(c), where the intensity of graphite-related de-lithiation peaks decreases proportionally. Therefore, despite the intrinsically low conductivity and diffusivity of silicon, the enhanced diffusivity observed in YNG-30% can be ascribed to electrode structural evolution. To further validate these results, additional resistance measurements and porosity comparisons were conducted to investigate the correlation between diffusion coefficient and overpotential.

### 3.5 Resistance

Here, coin full-cells were fabricated using the same cathode and anode electrode as employed in the pouch full-cells. After three initial formation cycles, the electrodes were divided into different SOC 10, 30, 50, 70, 90% and DOD 10, 30, 50, 70, 90%. At each state, various amplitudes of positive and negative pulse currents (ranging from 0.025 C to 10 C) were applied, and the corresponding potential responses were recorded. The correlations between the applied current and the potential shift were fitted using the Tafel equation, from which the exchange current density (*i*_0_) and interfacial charge-transfer resistance (*R*_ct_) were determined. The Tafel equations are expressed as follows:2
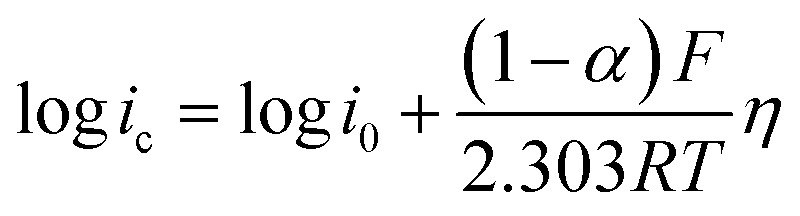
3
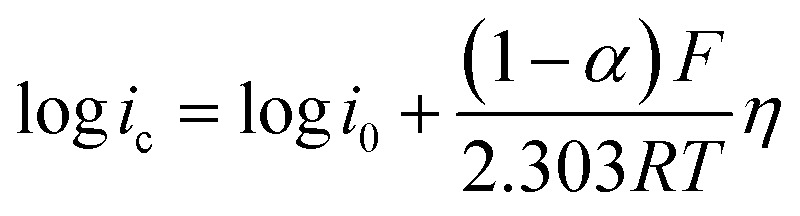
Here, *i*_c_ and *i*_a_ represent the cathodic and anodic current densities, respectively, normalized by the electrode surface area. *i*_0_ denotes the exchange current density, and *α* is the charge-transfer coefficient (assumed to be 0.5 for both cathodic and anodic reactions). *F* is the faraday constant, *T* is the absolute temperature, *R* is the gas constant, and *η* = (*E*_eq_ − *E*) is the overpotential. The *i*_0_ and *R*_ct_ can be correlated and calculated by the following relation:4
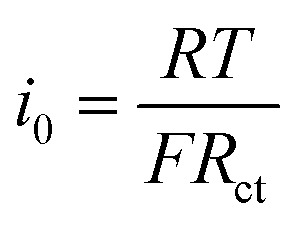
where *R*_ct_ is the charge-transfer resistance.

**Fig. 6:**
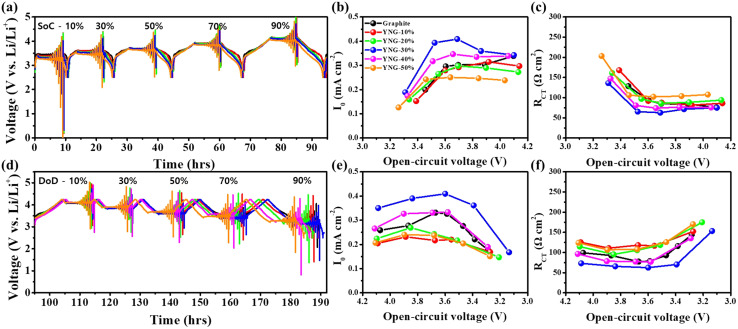
Pulse polarization process during (a) charging and (d) discharging. *i*_0_ and *R*_ct_ during charging (b and c), during discharging (e and f).

Fig. 6(a) and (d) present the pulse polarization process during charge and discharge, respectively, while (b) and (e) show the *i*_0_ and (c) and (f) display the *R*_ct_. The corresponding Tafel plots are provided in Fig. S5 and S6.

During both lithiation and de-lithiation, a large *R*_ct_ was observed at the low-voltage regions, namely the beginning of charging and the end of discharging, with lithiation exhibiting a characteristic ‘L-shaped’ profile. This behavior arises because electrochemical reactions inside the electrode are insufficiently activated at low SOC.^46^ As lithiation proceeds, the reactions become more active, the electrode potential increases, and interparticle contact improves, resulting in a decrease in *R*_ct_.^41^ A similar but symmetric trend was also observed during de-lithiation. Such features are common to both graphite and silicon electrodes. However, the effect is more pronounced for Si owing to its intrinsically slower Li^+^ insertion/extraction kinetics compared to graphite.^53^ Comparison among the YNG electrodes revealed the following order of *R*_ct_: YNG-30 < YNG-40 < YNG-20 < YNG-10 < YNG-50. In general, higher Si content is expected to increase *R*_ct_ owing to the low conductivity and sluggish reaction kinetics of Si; however, the experimental results indicate that additional factors are involved.^30^

**Fig. 7:**
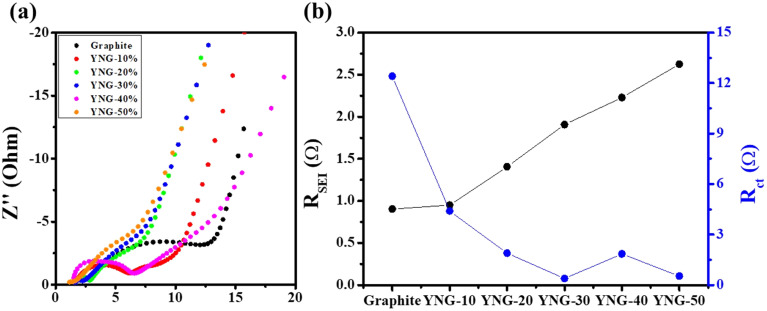
(a) Nyquist plots and (b) corresponding resistance components (*R*_SEI_ and *R*_ct_) of graphite and YNG composite electrodes with varying Si content, measured after three formation cycles at a fully discharged state.

To further clarify these differences, EIS measurements were conducted after three formation cycles to quantitatively separate each resistance component. This approach was adopted because, in composite electrodes, factors such as the binder, internal structural evolution, and SEI formation can contribute to the overall impedance. While the pulse polarization method is employed to evaluate the *R*_ct_, it is difficult to fully exclude the influence of volume expansion and SEI growth in high-silicon electrodes. Therefore, EIS measurements were performed to confirm the results. As shown in Fig. 7(b) and Table. S2, the *R*_SEI_ was lowest for the graphite electrode and increased proportionally with higher silicon content, indicating that greater silicon-induced expansion can lead to a thicker or more heterogeneous SEI layer. In contrast, the *R*_ct_ was highest for graphite and gradually decreased with increasing silicon content. Compared to silicon, graphite exhibits high conductivity and stable electrochemical properties. Considering that the initial electrode conditions were nearly identical, this indicates that changes in the electrode structure are the cause. This trend is found to be strongly correlated with the electrode porosity. According to Yu Wang *et al.*, the degree of calendering in graphite electrodes significantly affects both resistance and rate performance, where lower porosity electrodes exhibit higher *R*_ct_. This occurs because higher porosity allows the electrolyte to permeate uniformly throughout the electrode, whereas lower porosity restricts electrolyte infiltration and increases the tortuosity of Li^+^ transport pathways.^54^

### 3.6 Porosity & rate performance

As mentioned earlier, the irreversible expansion occurring after the formation cycles can alter the porosity of the electrode. The porosity values were calculated based on the mass-thickness-density relationship, as expressed by the following equations:^29,54^5
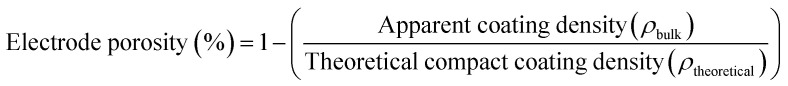
6
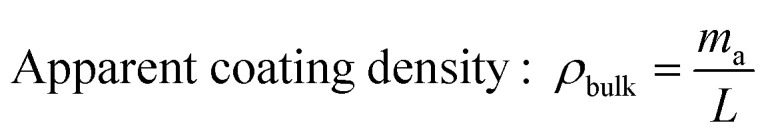
7
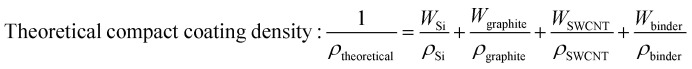
Here, *m*_a_ is the areal mass loading of the coated layer, *L* is the electrode thickness measured by *in situ* dilatometry, and *W*_i_ and *ρ*_i_ represent the mass fraction and true density of each component (graphite, Si-alloy, binder, and conductive additive). The theoretical density (*ρ*_theoretical_) of the composite electrode was calculated according to the rule of mixtures based on these measured values. When irreversible expansion increases the electrode thickness from *L*_0_ to L′ while the areal mass remains constant, the updated porosity is obtained as:8
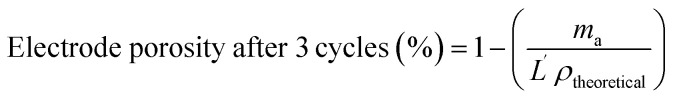


**Fig. 8:**
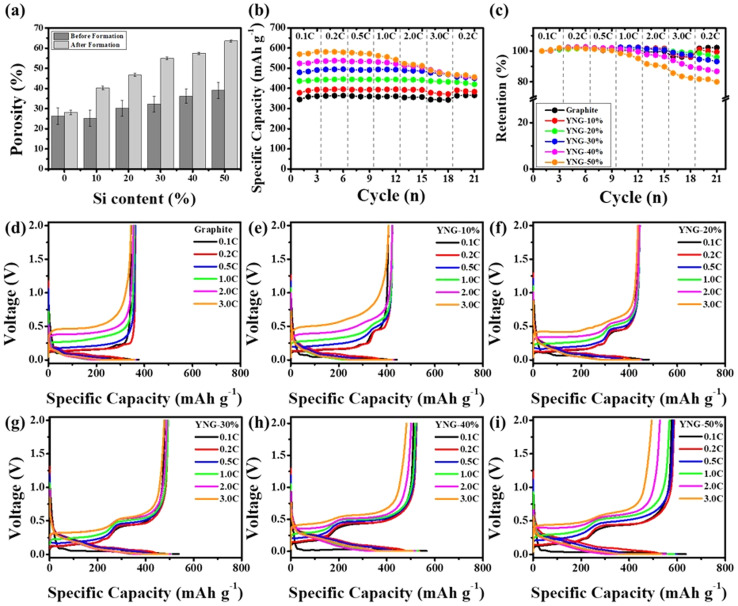
(a) Porosity evolution after formation cycling; (b) rate performances and (c) capacity retention at different current densities; charge–discharge profiles at 0.1–3.0 C for (d) graphite, (e) YNG-10%, (f) YNG-20%, (g) YNG-30%, (h) YNG-40%, and (i) YNG-50%.

As shown in Fig. 2(b), the degree of irreversible expansion increases with higher silicon content, which, according to the calculation parameters in Table S3, leads to the porosity increase presented in Fig. 8(a). This corresponds to the expansion of channels, defined as sufficiently large pores that allow many solvated Li^+^ to migrate freely together with the electrolyte.^55^ Nevertheless, the enlarged surface area resulting from higher porosity, together with the repeated expansion and contraction of silicon during cycling, promotes continuous SEI growth and electrolyte decomposition, leading to an increase in *R*_SEI_. For the graphite electrode, the porosity remained nearly constant and low, increasing only slightly from 26.3% to 28.1% owing to its small irreversible expansion. In contrast, the YNG-50% electrode exhibited the largest increase in porosity, rising from 39.1% to 63.6% as a result of significant irreversible expansion. Accordingly, the EIS results reconfirm that the high *R*_ct_ of the graphite electrode originates from its low porosity. In contrast, the YNG-50% electrode exhibited a decrease in *R*_ct_ due to the higher porosity induced by significant irreversible expansion, although such expansion also strongly affected SEI formation and growth. The YNG-30% electrode, which exhibited the best diffusivity and the lowest resistance, achieved a balance between silicon content and porosity, resulting in both low *R*_SEI_ and *R*_ct_ values and thus improved performance in both thermodynamic and kinetic aspects.

Based on the analysis of resistance, *D*_Li^+^_, and overpotential, 2016-type half-cells were assembled using graphite and YNG electrodes, and their rate performance was evaluated under current densities ranging from 0.2 to 3.0 C. At 3 C, the capacity retentions of the graphite, YNG-10%, YNG-20%, YNG-30%, YNG-40%, and YNG-50% electrodes were 95.1%, 94.5%, 97.8%, 95.5%, 88.1%, and 80.2%, respectively. As shown in Fig. 8(f), the YNG-30% electrode exhibited the lowest overpotential at 3 C, which can be attributed to enhanced diffusivity and charge-transfer resulting from the optimized porosity. However, despite the lower overpotential of the YNG-30% electrode compared to YNG-20%, its capacity retention was slightly lower, as revealed by the recovery behavior upon reducing the C-rate back to 0.2 C, indicating degradation of silicon. Such degradation is mainly attributed to the repeated formation and growth of the SEI layer caused by the continuous expansion and contraction of silicon during cycling, as confirmed by the EIS results. In addition, these rate performance results are consistent with the trends reported by Eunchae Kim *et al.* for graphite/silicon composite anodes, further demonstrating that electrodes containing silicon can also be advantageous for high-rate charge/discharge performance even in high-density electrodes fabricated by calendaring.^56^ In summary, the enhancement in the performance of the rate can be ascribed to the porosity that is formed by the irreversible expansion. This porosity provides channels for the migration of solvated Li^+^, promotes interparticle diffusion in graphite, increases the electrolyte storage capacity, and consequently reduces the *R*_ct_. This, in turn, enhances interfacial diffusion.

## 4 Conclusion

In this study, the thermodynamic and kinetic behaviors of graphite/silicon alloy composite anodes were analyzed using a three-electrode pouch cell and *in situ* dilatometry. The *D*_Li^+^_ was determined by GITT while accounting for electrode thickness variation, and the resistance components were quantified by pulse polarization and EIS. This integrated approach provides an effective framework for quantitatively evaluating the influence of irreversible expansion on electrode structure and electrochemical performance.

**Fig. 9:**
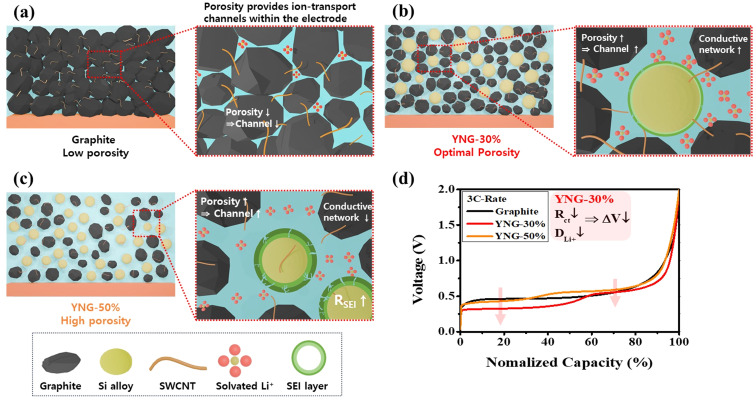
Schematic illustration of the correlation between silicon content, electrode porosity, and electrochemical characteristics: (a) graphite, (b) YNG-30%, (c) YNG-50%, and (d) comparison of overpotential in normalized capacity at 3 C-rate.

Electrochemical analysis revealed distinct structural–electrochemical correlations among the electrodes. The graphite electrode underwent only minor irreversible expansion (∼2.5%) after formation. It maintained a dense, low-porosity structure (26.3–28.1%), which restricted Li^+^ transport and led to a relatively high *R*_ct_. In contrast, the YNG-30% electrode experienced pronounced structural relaxation during the formation process, with its porosity increasing from 32.3% to 55.0%. This relaxation reduced *R*_ct_ and improved both interparticle and interfacial diffusion, facilitating the transport of solvated Li^+^ ions and leading to a lower overpotential in the graphite reaction region. However, the YNG-50% electrode underwent excessive expansion accompanied by SEI thickening, which increased *R*_SEI_ and deteriorated Li^+^ diffusivity, resulting in higher overpotential. Consequently, the YNG-30% electrode achieved an optimal balance between porosity, SEI stability, and internal structure, exhibiting high diffusivity, low overpotential, and superior rate capability. These correlations are schematically illustrated in Fig. 9.

Although irreversible expansion is generally regarded as a degradation factor, the results demonstrate that when properly controlled it can increase porosity, reduce internal resistance, and improve both interfacial and interparticle diffusion in the graphite staging region. However, the Si-dominant region still exhibited low diffusivity and high overpotential, confirming the intrinsic kinetic limitation of silicon. Irreversible expansion may also penalize volumetric energy density. Overall, these findings suggest that graphite/silicon composite anode design should simultaneously optimize silicon content, SEI stability, and porosity, to achieve a balanced trade-off between fast-charging/high-power capability and volumetric energy density.

## Author contributions

Min-ho Lee: writing – original draft, methodology, investigation, formal analysis. Orynbassar Mukhan: software, methodology, data curation, formal analysis. Carlos Tafara Mpupuni: software, investigation, visualization. Batukhan Tatykayev: validation, data curation, software. Zhumabay Bakenov: writing – review & editing, visualization, supervision. Sung-Soo Kim: writing – review & editing, visualization, supervision, funding acquisition, conceptualization, resources.

## Conflicts of interest

There are no conflicts of interest to declare.

## Supplementary Material

RA-015-D5RA07317K-s001

RA-015-D5RA07317K-s002

RA-015-D5RA07317K-s003

RA-015-D5RA07317K-s004

RA-015-D5RA07317K-s005

RA-015-D5RA07317K-s006

RA-015-D5RA07317K-s007

RA-015-D5RA07317K-s008

## Data Availability

Data will be made available upon request. All relevant data to support this research can be found in the article and the supplementary information (SI). The Supplementary Information includes additional electrochemical data, electrode conditions, and density information of the materials. See DOI: https://doi.org/10.1039/d5ra07317k.
