# Supplementary figures and images for: The impact of volume expansion on thermodynamic and kinetic properties of graphite/Si alloy composite anodes
Source: RSC Adv. 2025 Dec 4;15(56):47790–802. doi: 10.1039/d5ra07317k (PMC12679607; doi:10.1039/d5ra07317k)

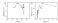

Supplement: RA-015-D5RA07317K-s003 [file RA-015-D5RA07317K-s003.pdf]

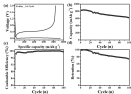

Supplement: RA-015-D5RA07317K-s004 [file RA-015-D5RA07317K-s004.pdf]

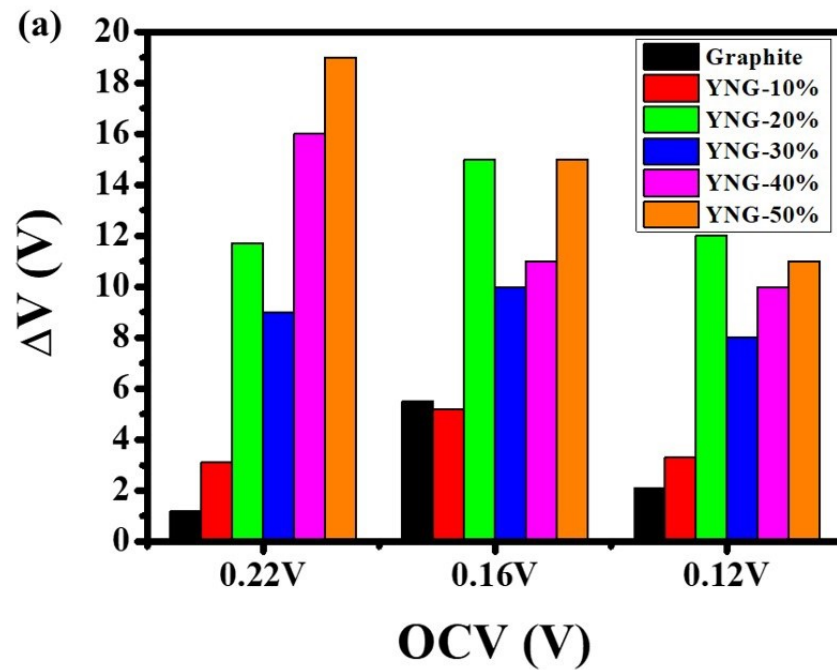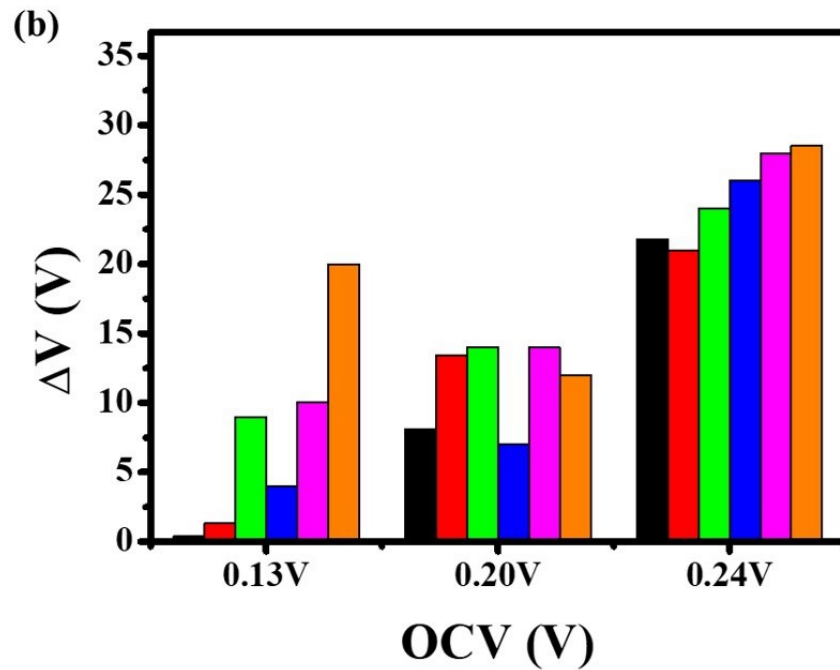

Supplement: RA-015-D5RA07317K-s005 [file RA-015-D5RA07317K-s005.pdf]

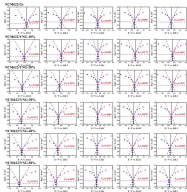

Supplement: RA-015-D5RA07317K-s006 [file RA-015-D5RA07317K-s006.pdf]

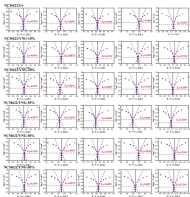

Supplement: RA-015-D5RA07317K-s007 [file RA-015-D5RA07317K-s007.pdf]
