# Supplementary material for: The impact of volume expansion on thermodynamic and kinetic properties of graphite/Si alloy composite anodes
Source: RSC Adv. 2025 Dec 4;15(56):47790–802. doi: 10.1039/d5ra07317k (PMC12679607; doi:10.1039/d5ra07317k)
Supplement: RA-015-D5RA07317K-s002 [file RA-015-D5RA07317K-s002.pdf]

Supplementary Information (SI) for RSC Advances.
This journal is © The Royal Society of Chemistry 2025
