# Supplementary material for: The impact of volume expansion on thermodynamic and kinetic properties of graphite/Si alloy composite anodes
Source: RSC Adv. 2025 Dec 4;15(56):47790–802. doi: 10.1039/d5ra07317k (PMC12679607; doi:10.1039/d5ra07317k)
Supplement: RA-015-D5RA07317K-s008 [file RA-015-D5RA07317K-s008.pdf]

Supplementary Information (SI) for RSC Advances.
This journal is © The Royal Society of Chemistry 2025

The Impact of Volume Expansion on Thermodynamic and Kinetic Properties of Graphite/Si Alloy Composite Anodes

Min-ho Lee[a], Orynbassar Mukhan[b], Carlos Tafara Mpupuni[a], Batukhan Tatykayev[b,c], Zhumabay Bakenov[c,d], and Sung Soo Kim[a,*]

[a] *Graduate School of Energy Science and Technology, Chungnam National University, 99 Daehak-ro, Yuseong-gu, Daejeon 34134, Republic of Korea.*

[b] *Institute of Batteries LLC, Kabanbay batyr Ave. 53, Astana, 010000, Kazakhstan*

[c] *National Laboratory Astana, Nazarbayev University, Kabanbay Batyr Ave. 53, Astana, 010000, Kazakhstan*

[d] *Department of Chemical and Materials Engineering, School of Engineering and Digital Sciences, Nazarbayev University, Kabanbay Batyr Ave. 53, Astana, 010000, Kazakhstan*

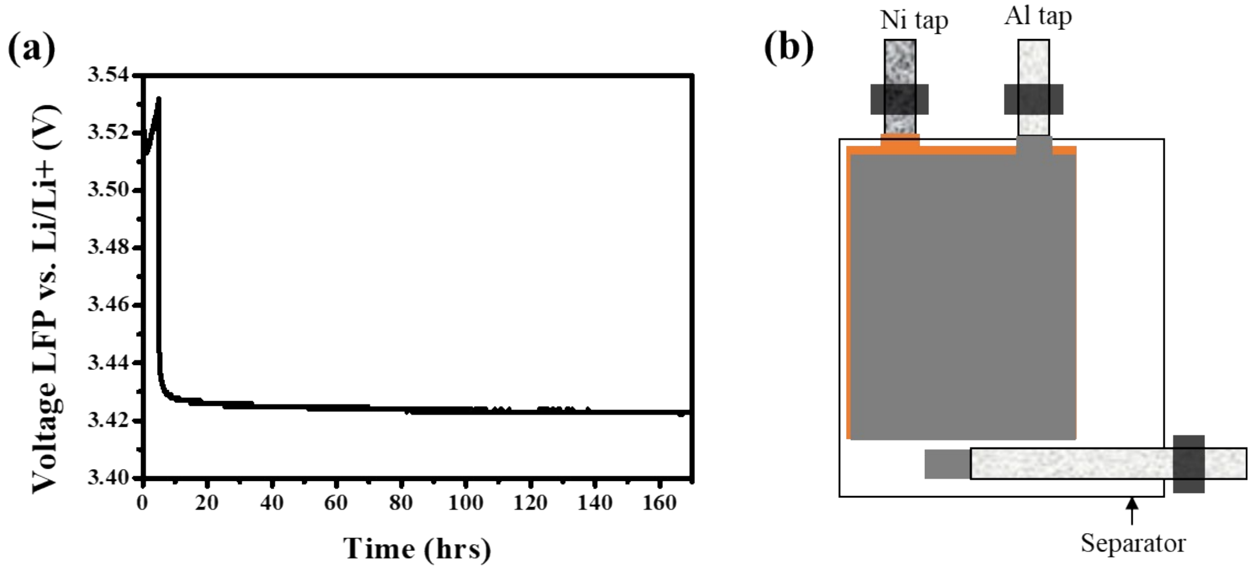

Fig. S1 (a) Voltage profile of the LFP electrode after charging at 0.1C for 5 h, demonstrating stable performance as a reference electrode. (b) Schematic illustration of the three-electrode pouch cell.

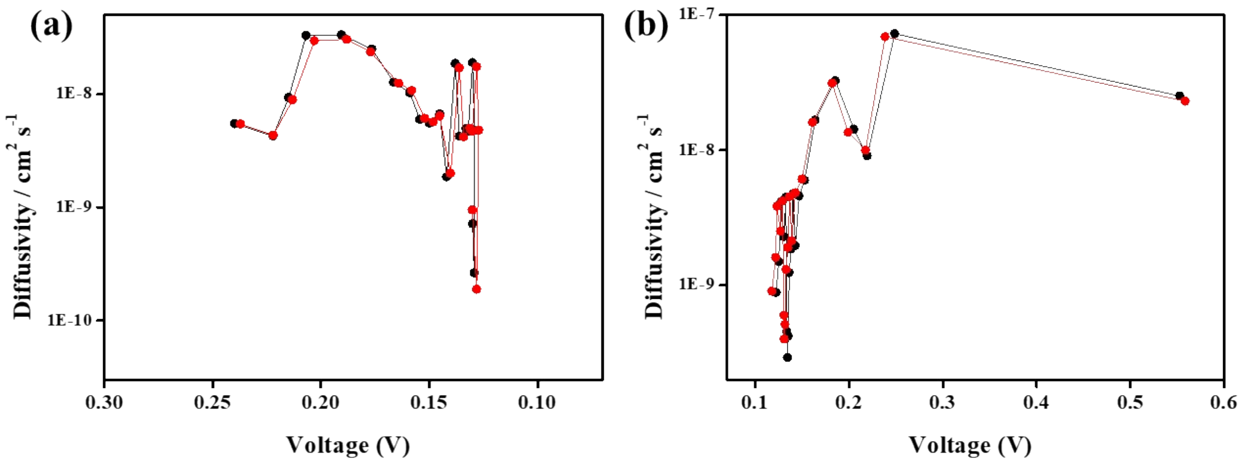

Fig. S2 Reproducibility test of graphite electrodes under identical conditions: (a) lithiation and (b) de-lithiation processes showing consistent potential profiles and diffusivity trends (deviation within 10 %).

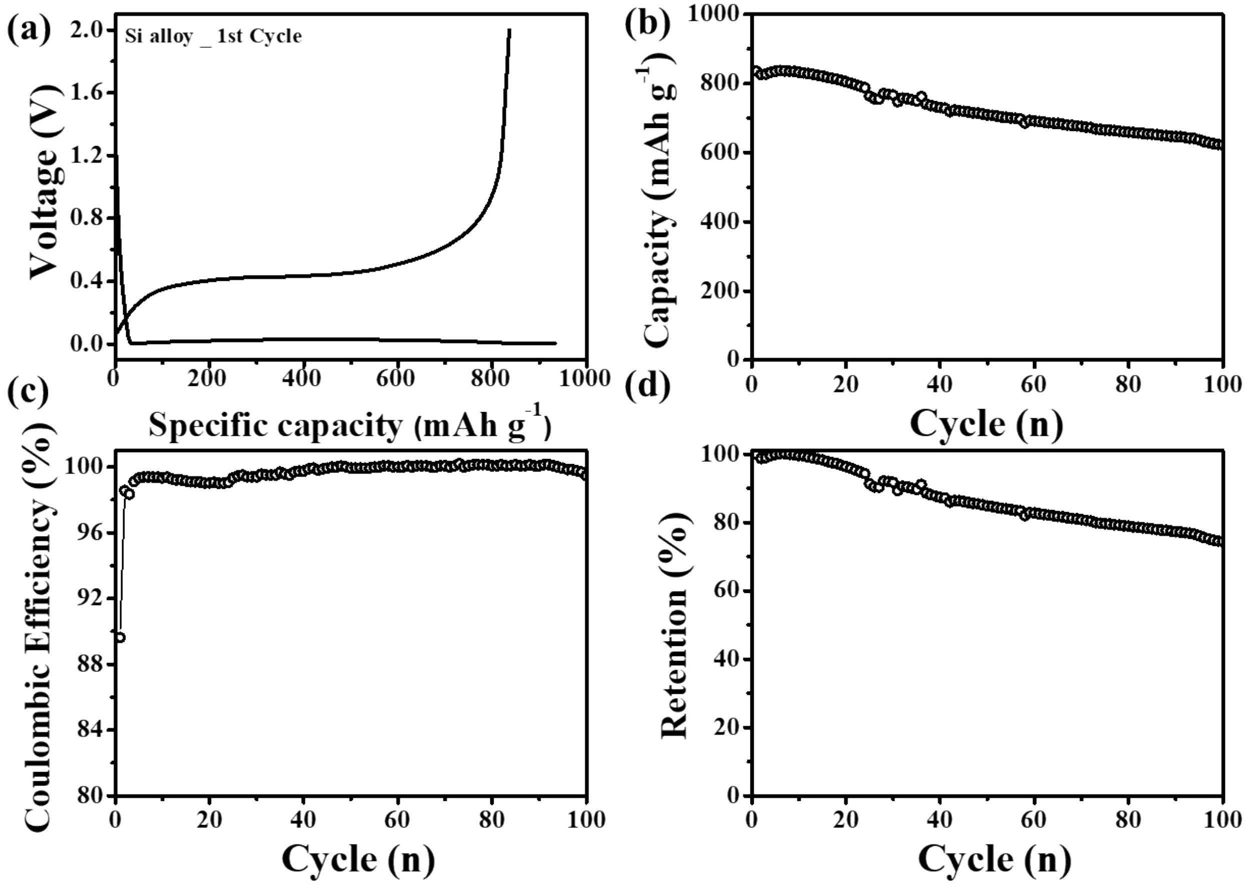

Fig. S3 Electrochemical performance of the Si alloy anode. (a) initial voltage profile, (b) cycling performance, (c) coulombic efficiency, and (d) capacity retention.

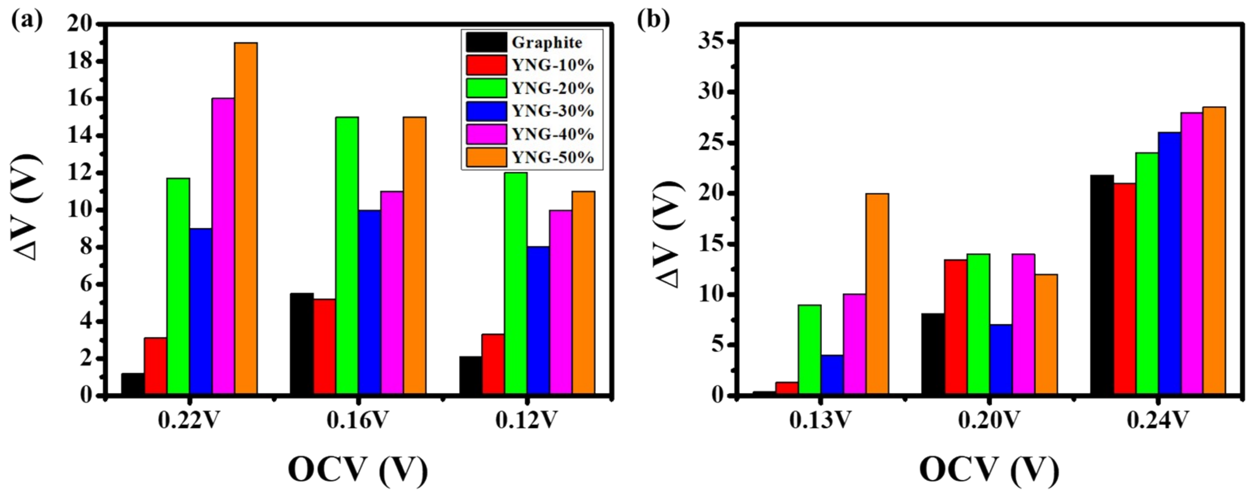

Fig. S4 ΔV as a function of OCV during (a) lithiation and (b) de-lithiation.

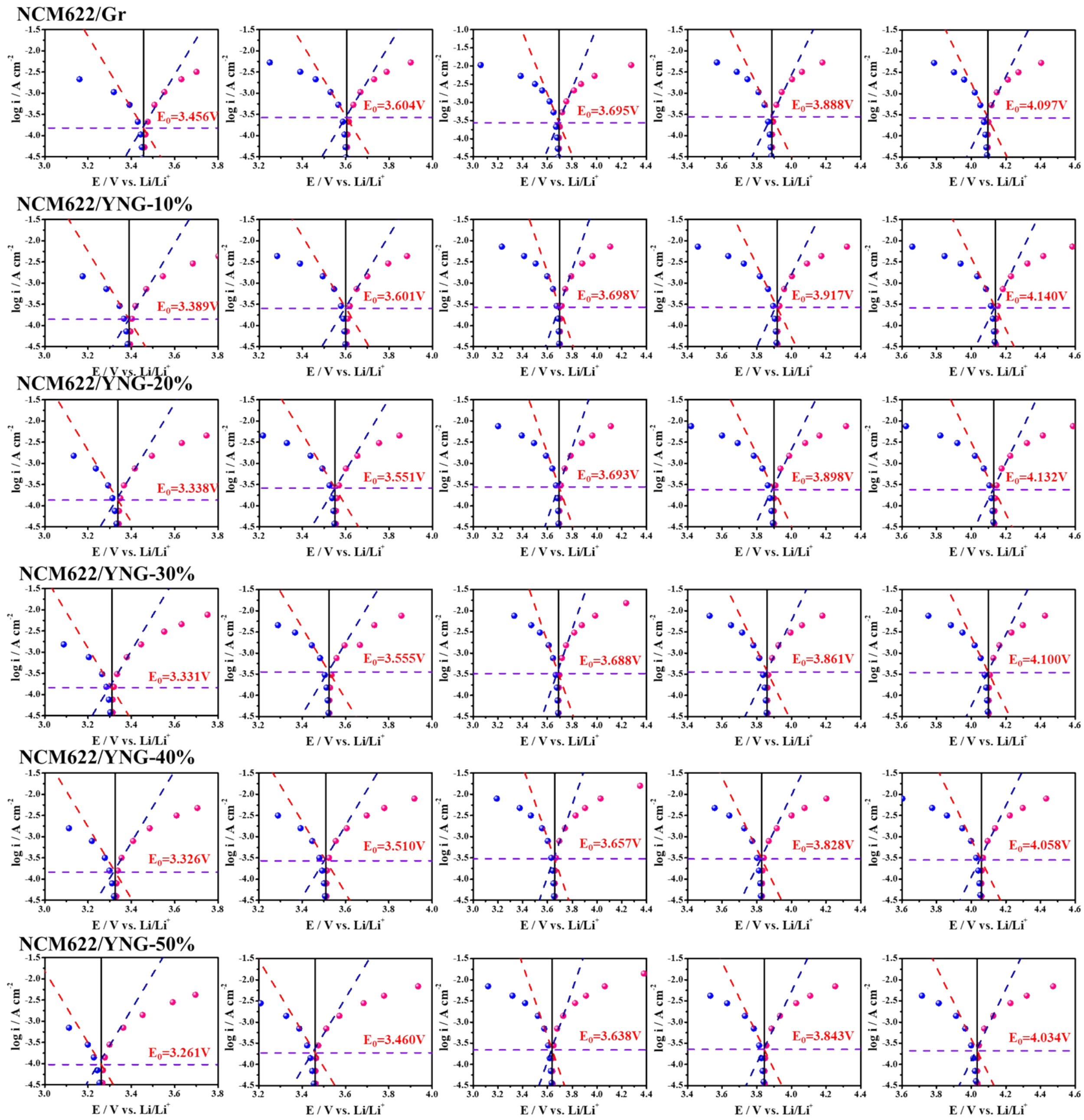

Fig. S5 Tafel plots of the graphite/Si alloy anode at different states of charge (SOC, from left to right: 10, 30, 50, 70, and 90 %).

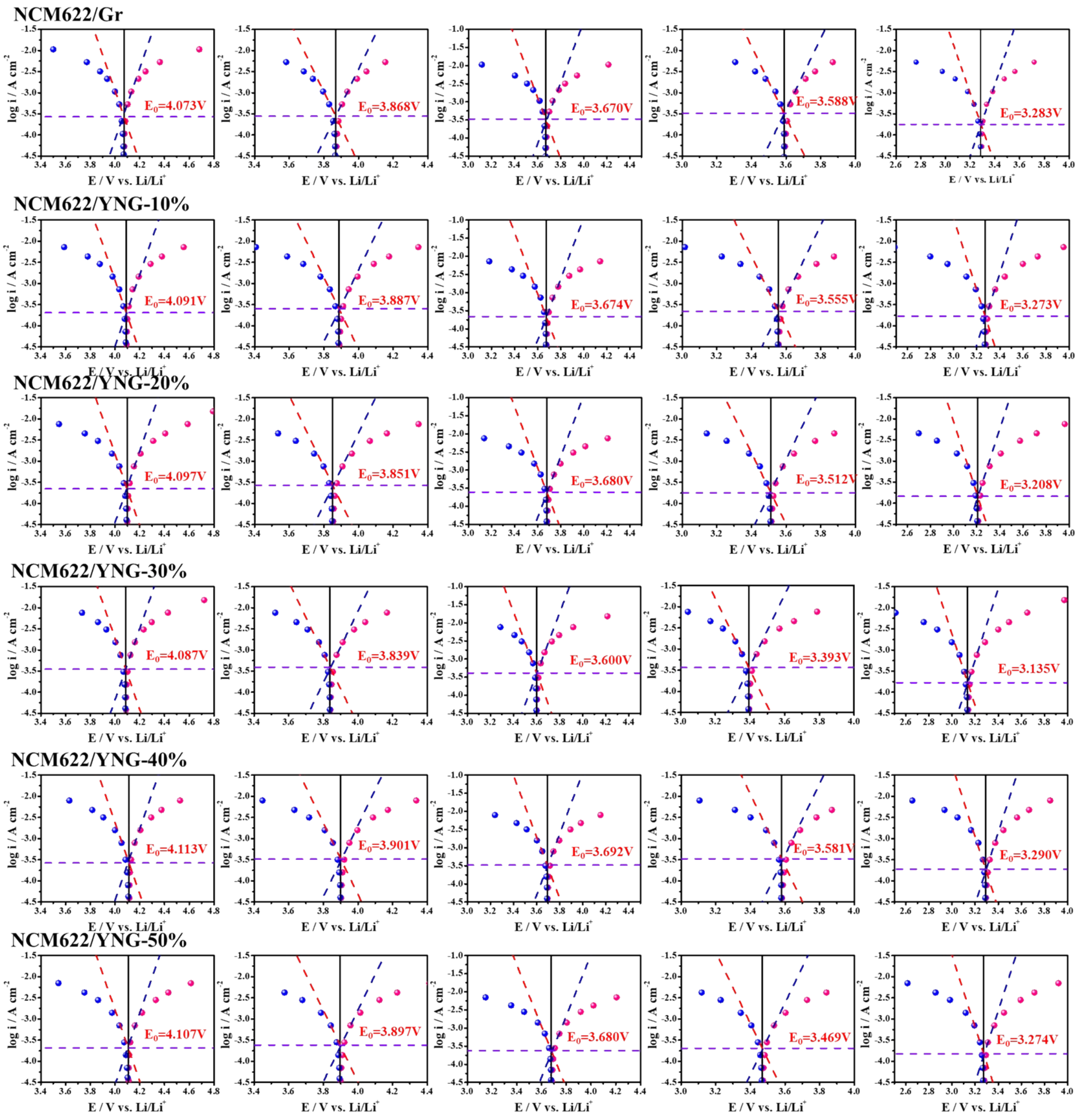

Fig. S6 Tafel plots of the graphite/Si alloy anode at different depths of discharge (DOD, from left to right: 10, 30, 50, 70, and 90 %).

Table. S1 Electrode properties of graphite, and graphite/Si composite anodes.

Anode	Graphite content (wt%)	Si alloy content (wt%)	Electrode Density (g cc^{-1})	Mass loading (mg cm^{-2})	Initial Thickness (μm)
**Graphite**	100	0	1.60	3.84	24
**YNG-10%**	90	10	1.68	4.20	25
**YNG-20%**	80	20	1.62	4.38	27
**YNG-30%**	70	30	1.63	4.08	25
**YNG-40%**	60	40	1.60	4.43	28
**YNG-50%**	50	50	1.58	3.96	25

Table. S2 Equivalent circuit fitting results for EIS analysis.

Anode	OCV (V)	R_S (Ω)	R_{SEI} (Ω)	R_{ct} (Ω)
**Graphite**	2.71	1.63	0.90	12.4
**YNG-10%**	2.83	1.84	0.95	4.39
**YNG-20%**	2.80	1.63	1.41	1.89
**YNG-30%**	2.80	1.63	1.91	0.38
**YNG-40%**	2.81	1.52	2.23	1.85
**YNG-50%**	2.82	1.15	2.63	0.53

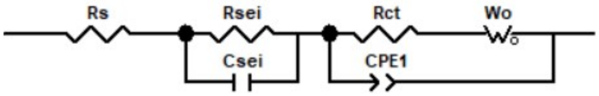

Table. S3 Parameters used for porosity calculation.

Parameter	Value
ρ(Graphite) g cm^{-3}	2.22
ρ(Si-alloy) g cm^{-3}	3.41
ρ(SWCNT) g cm^{-3}	1.3
ρ(CMC) g cm^{-3}	1.59
ρ(SBR) g cm^{-3}	0.94
$Porosity\ (\%) = 1 - \frac{electrode\ density}{theoretical\ density} \times 100$	
